# Gender differences in perceived environmental correlates of physical activity

**DOI:** 10.1186/1479-5868-2-12

**Published:** 2005-09-13

**Authors:** Enrique Garcia Bengoechea, John C Spence, Kerry R McGannon

**Affiliations:** 1 Alberta Centre for Active Living, Faculty of Physical Education and Recreation E-424 Van Vliet, University of Alberta, Edmonton, Alberta, Canada; 2 Faculty of Physical Education and Recreation, E-424 Van Vliet, University of Alberta, Edmonton, Alberta, Canada; 3 Department of Health and Sport Studies, University of Iowa, Iowa City, USA

## Abstract

**Background:**

Limited research has been conducted on gender differences in perceived environmental correlates of physical activity (PA). The purpose of this study was to explore the potential role of gender in the link between perceived environment and PA.

**Methods:**

Using a telephone-administered survey, data was collected on leisure time physical activity (LTPA), perceptions of the neighbourhood environment, and self-efficacy in a representative sample of 1209 adults from the province of Alberta, Canada. LTPA was regressed on ten measures of perceived neighbourhood environment and self-efficacy in a series of logistic regressions.

**Results:**

Women were more likely than men to perceive their neighbourhood as unsafe to go for walks at night (χ^2 ^= 67.46, p < 0.001) and to report seeing people being active in their neighbourhood (χ^2 ^= 6.73, p < 0.01). Conversely, women were less likely to perceive easy access to places for PA (χ^2 ^= 11.50, p < 0.01) and availability of places to buy things within easy walking distance from home (χ^2 ^= 4.30, p < 0.05). Adjusting for age, education, income, and place of residence, access to places for PA (OR = 2.49) and interesting things to look at in the neighbourhood (OR = 1.94), were associated with higher levels of LTPA in men. Access to places for PA (OR = 2.63) and reporting seeing people being active (OR = 1.50) were associated with increased LTPA among women. After controlling for sociodemographic variables and self-efficacy, the presence of shops and places to buy things within easy walking distance from home (OR = 1.73), interesting things to look at in the neighbourhood (OR = 1.65), and access to places for PA (OR = 1.82) were associated with higher levels of LTPA in men. Among women, no significant relationships were observed between perceived environment and LTPA after adjusting for self-efficacy.

**Conclusion:**

The results provide additional support for the use of models in which gender is treated as a potential moderator of the link between the perceived environment and PA. Further, the results suggest the possibility of differential interventions to increase PA based on factors associated with gender.

## Background

A growing body of scientific evidence has brought to public attention the negative effects of physical inactivity on health and the benefits of a physically active lifestyle [1,2]. Despite the well-documented physical, psychological, and social benefits of regular physical activity (PA) [3,4], physical inactivity remains pervasive. It is estimated that upwards of two-thirds of the industrialized world does not achieve minimum PA guidelines [5,2]. Physical inactivity thus constitutes a major public health concern [2] with related social and economic costs [6,7].

In an effort to solve the PA participation problem, research in the past two decades has employed theoretical perspectives to better understand the factors that enhance and detract from PA participation. In particular, social cognitive models that emphasize the interaction of intrapersonal factors, micro-environmental influences and PA behavior, have gained empirical support. This has allowed researchers to identify cognitive (e.g., efficacy beliefs), affective, and social influences on the individual's choice to be active and to aid in the development of interventions to increase PA levels [8]. Despite being identified as contributing toward behavior change, such individually-focused factors have generally been found to account for a modest proportion of the variance in PA behavior.

In recent years, it has been suggested that wider use of ecological models, which emphasize PA as being the result of multiple influences (i.e., intrapersonal, interpersonal, environmental, organizational and policy levels), hold great promise for addressing the PA participation problem [8-10]. The foregoing is underscored by significant changes observed over the past 50 years in the North American environment which promote decreased PA at the level of the individual. For example, in the United States, while daily vehicle miles traveled per person increased by 224% between 1950 and 2000, the proportion of trips to work by walking decreased 71% between 1960 and 2000 [11]. Transport systems and urban design (e.g., number and quality of pathways and cycleways, availability of buses and bus stops) play an important role in supporting or discouraging PA [12]. Urban sprawl increases distances between differential land use so that roads are more available than cycling paths or sidewalks [13-15].

Consistent with an ecological perspective [9,16], researchers have attempted to document and understand how different aspects of the physical environment may influence the extent to which individuals are more or less physically active [17-19]. Distribution and quality of local sport and recreational facilities, community clubs and churches, as well as features of the physical environment have all been shown to be associated with PA participation [20,21].

Researchers have begun to use models that allow selected demographic and personal characteristics such as socioeconomic status [22,23], gender [24-26] and weight status [27] to act as potential moderators of the effect of the perceived environment rather than as confounding variables. This is also consistent with an ecological perspective, which posits that there are interactions among levels in the system linking individuals with their environments [9,16].

Understanding the role of gender in the relationship between the perceived environment and PA participation is of particular importance since women typically exhibit lower levels of PA than their male counterparts [24,28]. Research that has attempted to elucidate gender differences in PA determinants has also revealed that women may face different barriers (e.g., lack of time due to multiple roles, perceptions of safety, and environmental access) than men, which can limit their PA participation [29,30]. For instance, in a survey of 3,032 U.S. adults [16], psychological factors such as mastery, exercise self-efficacy, and perceived control over health explained educational differences in PA among women.

Despite the emerging interest in the role of the environment and PA participation, few studies have systematically explored differences between women and men in terms of correlates of PA in general, and environmental correlates in particular. Among the exceptions is a recent study [24] that prospectively examined associations of changes in perceptions of local environmental attributes with changes in neighbourhood walking. Results revealed contrasting findings for men and women who reported traffic as less of a problem, with men being 61% less likely to increase walking. Women, on the other hand, were 76% more likely to have done so. Another finding was that men reporting positive changes in aesthetics and convenience were twice as likely to increase their walking. Women who reported positive changes in convenience were more than twice as likely to have increased their walking. In a related study [25], neighborhood walking was associated with high ratings of "aesthetics", "convenience" of facilities and "access" to facilities in men, and with high ratings of "convenience" in women. "Access", on the other hand, was negatively associated with neighborhood walking in women. Finally, in one recent study examining the role of gender on the link between PA and the perceived environment [26], perceptions of safety during day time and convenience (e.g., having shops within walking distance) were positively associated with walking among women. Having a park/open space within walking distance was the only environmental dimension associated with walking in men. Furthermore, there was evidence of substantial differences in perceptions of the environment between genders for both low and high walking groups.

These studies contribute to the literature that documents significant cross-sectional associations of perceived environmental attributes with PA [31]. However, more studies are needed to further elucidate relevant gender differences in PA participation [24]. In particular, little is known yet about how gender may interact with perceptions of the environment in order to influence PA participation. For instance, in relation to PA, it is likely self-efficacy beliefs vary by gender [16] and thus may account for much of any gender differences in perceived environment. Furthermore, no studies have been published on how perceptions of neighbourhood environment relate to PA in Canada. Consequently, the purposes of this study were to determine: a) if gender differences in perceptions of the physical environment exist; b) how these differences may have an impact on leisure time physical activity (LTPA) patterns of individuals in a representative sample of adults from the Province of Alberta; and c) if these gender differences are explained by PA self-efficacy.

## Methods

### Design and Sample

Participants included a representative sample of 1,209 adults (Males = 600; Females = 609) aged 18 years and over residing in the Province of Alberta, Canada. Data were collected by telephone interview between October 29, 2002, and December 1, 2002. Three separate subsamples represented Edmonton, Calgary, and the rest of the province. A random-digit dialling approach ensured that respondents had an equal chance of being contacted whether or not their household was listed in a telephone directory. Approximately 54% of the total number of valid households responded to the survey. A random sample of 1,209 is considered accurate within +/- 2.8, 19 times out of 20.

### Measures

#### Leisure-Time Physical Activity

The Godin Leisure-Time Exercise Questionnaire [32] was used to estimate LTPA levels. Self-reported weekly frequencies of strenuous, moderate, and light activities were multiplied by their estimated value in METs (nine, five, and three respectively). Total weekly LTPA was calculated by adding the products of the separate components. Albertans were considered sufficiently physically active if they expended 38 METs a week for men or 35 METs a week for women. These cutoff values were derived based upon the work of several researchers [33-35]. According to Jacobs et al. [34], these values equal 300–400 MET-minutes per day. This number of MET-minutes is equivalent to a weekly energy expenditure of 2,000 kcals [33]. An energy expenditure of 2,000 kcals or more per week is associated with a reduced risk of heart disease [35]. Based on this criterion, we created a dichotomous variable for LTPA: inactive or active.

#### Perceived environment

Nine questions from the International Physical Activity Prevalence Study Environmental Survey Module [36] were used to assess respondents' perceptions of neighbourhood characteristics that may influence LTPA. The module consists of a series of items that reflect current thinking in assessment of environmental factors for walking and bicycling in one's neighbourhood and for which reliability and validity have been assessed [36]. Since walking (82%) is the primary source of LTPA among Canadians and bicycling is reported by a significant proportion (45%) of the population [37], the environment scale was deemed to be an appropriate measure of neighbourhood influences on LTPA.

Specifically, respondents were asked to indicate how much they agree or disagree with the following items, on a 4-point scale where 1 means strongly disagree and 4 means strongly agree: "Many shops, stores, or other places to buy things I need are within easy walking distance of my home"; "There are sidewalks on most of the streets in my neighbourhood"; "In and around my neighbourhood, there are facilities for bicycling such as special bicycle lanes, bicycle paths, and shared use trails for cyclists and pedestrians"; "My neighbourhood has several free or low cost recreation facilities, such as parks, walking trails, bike paths, playgrounds, and recreation centres"; "The crime rate in my neighbourhood makes it unsafe to go for walks at night"; "There is so much traffic on the streets that it makes it difficult or unpleasant to walk in my neighbourhood"; "I see many people engaging in physical activity in my neighbourhood doing things like walking, jogging, cycling, or playing sports and active games"; "There are many interesting things to look at while walking in my neighbourhood." In addition, respondents were asked to rate on the same 5-point scale their agreement with the following statement: "I have easy access to places where I can get physical activity." For the purposes of this study, the responses to each of the environmental variables were collapsed into categories of disagree (1 or 2) or agree (3 or 4).

#### Sociodemographic

Sociodemographic variables included age (coded as continuous variable), education (less than secondary, secondary, post-secondary), annual household income (< $30,000;$30,000–60,000; > $60,000), and location (urban, rural).

#### Self-Efficacy

Based upon previous research [38], a self-efficacy score was derived by averaging the respondents' ratings on three items assessing their confidence in overcoming, respectively, fatigue, bad weather, and time constraints to participating in moderate LTPA. The Cronbach's alpha coefficient for these three items was 0.77.

### Statistical Analyses

All data were cleaned and edited following standard quality control procedures. The analyses were conducted with the Statistical Package for the Social Sciences (SPSS), version 13.0. Data were weighted to account for the circumstance that the sample sizes of completed interviews for Edmonton, Calgary, and other Alberta, were not proportional to the Alberta population. Specifically, the area samples were multiplied by a weighting factor to account for the slight over representation of residents from Edmonton and Calgary and the slight under representation of residents from other parts of Alberta. However, according to the index of dissimilarity [39], little difference existed between the age groupings of our sample and that of the Alberta population [40]. In addition, the proportion of men and women in our sample was very similar to that of the Canadian and Alberta populations [40]. Thus, the sample adequately reflected the population from which it was drawn.

Descriptive statistics were calculated for sociodemographic variables, self-efficacy, and LTPA. Differences between women and men were tested with chi-square analysis, in the case of categorical variables, and *t *tests in the case of continuous variables. Gender differences in responses to each environmental item were tested with chi-square analysis. Because PA scores are typically not normally distributed [41], non-parametric procedures were adopted to analyse the LTPA data. Specifically, unconditional logistic regression analyses, with LTPA as the dependent variable, were used to calculate unadjusted and adjusted ORs and the 95% CIs for each environmental variable. To calculate the adjusted ORs, LTPA was regressed on the sociodemographic variables (age, education, income, and location) and, then, self-efficacy was entered on the second step. The decision about which variables to include in the regression analysis was guided by ecological models of PA [9,16] and other research showing relations between sociodemographic variables and LTPA [42-44]. Conducting separate analyses for males and females allowed us to examine unique patterns of association between the environmental variables and LTPA.

## Results

Based on the cut-off values described earlier, 59.9% of men and 54.2% of women were considered physically active. As Table 1 also shows, men displayed significantly higher levels of self-efficacy (*t *= 6.02, p < 0.001) and had higher annual household income than women (χ^2 ^= 43.46, p < 0.001). Men were also slightly younger than women (*t *= -2.33, p < 0.05). As seen in Table 2, women were more likely than men to perceive their neighbourhood as unsafe to go for walks at night (χ^2 ^= 67.46, p < 0.001) and to report seeing people being active in their neighbourhood (χ^2 ^= 6.73, p < 0.01). Conversely, women had lower perceptions of easy access to places for PA (χ^2 ^= 11.50, p < 0.01) and of availability of places to buy things within easy walking distance from home (χ^2 ^= 4.30, p < 0.05).

**Table 1:** Characteristics of Participants

	Male (n = 600)	Female (n = 609)	χ^2 ^or t
Age			
Mean	41.4	43.6	-2.33*
SD	15.4	16.2	

Education (% in each category)			0.96
Less than high school	12.9	11.8	
High school	19.7	21.7	
Postsecondary	67.4	66.5	

Income (% in each category)			43.46***
<$30,000	9.3	23.3	
$30,000–60,000	30.9	34.9	
>$60,000	59.8	41.8	

Location (% in each category)			0.64
Urban	81.7	83.4	
Rural	18.3	16.6	

Self-efficacy			
Mean	7.3	6.5	6.02***
SD	2.3	2.4	

LTPA (% active)	59.9	54.2	4.16*

* p < .05., ***p < 0.001

**Table 2:** Gender Differences in Perceptions of the Physical Environment

	Male		Female			
	Number	%	Number	%	χ^2^	(df, n)

Shops					4.30*	1, 1205
Disagree	202	33.8	240	39.5		
Agree	396	66.2	367	60.5		
Transit stop					2.09	1, 1178
Disagree	199	34.1	179	30.1		
Agree	385	65.9	415	69.9		
Sidewalks					0.57	1, 1176
Disagree	102	17.6	95	15.9		
Agree	478	82.4	501	84.1		
Free or low cost					0.01	1, 1198
Disagree	137	23.0	140	23.3		
Agree	459	77.0	462	76.7		
Crime					67.46***	1, 1178
Disagree	506	85.3	378	64.6		
Agree	87	14.7	207	35.4		
Traffic					0.71	1, 1195
Disagree	482	81.1	476	79.2		
Agree	112	18.9	125	20.8		
Seeing people					6.73**	1, 1196
active						
Disagree	170	28.6	133	22.1		
Agree	424	71.4	469	77.9		
Interesting things					0.26	1. 1203
Disagree	204	34.1	198	32.7		
Agree	394	65.9	407	67.3		
Easy access					11.50**	1, 1066
Disagree	76	14.2	118	22.2		
Agree	118	85.8	413	77.8		

* p < .05., **p < .01., ***p < 0.001.

The results revealed some interesting differences between men and women in terms of the perceived neighbourhood characteristics that are associated with an increased likelihood of being physically active. As table 3 shows, men who perceived many interesting things to look at while walking in their neighbourhood were 83% more likely to be physically active than those who did not. This association remained statistically significant even after adjusting for sociodemographic variables (OR = 1.94) and self-efficacy (OR = 1.65). When adjusting for sociodemographic variables and self-efficacy, perceptions of presence of shops and places to buy things within easy walking distance from home, were associated with increased LTPA among men as well (OR = 1.73). Among women, perceptions of availability of free or low cost recreational facilities were linked with higher levels of LTPA (OR = 1.53). However, this relationship did not remain statistically significant after adjusting for sociodemographic variables and self-efficacy. Seeing many people engaging in PA in their neighbourhood also increased the likelihood of being physically active in women (OR = 1.50). This association remained statistically significant after adjusting for sociodemographic variables (OR = 1.78) but not after self-efficacy was included in the model.

**Table 3:** Perceived Environmental Correlates of Being Active Versus Inactive

		Male			Female	
	Unadjusted OR (95% CI)	Adjusted^1 ^OR (95% CI)	Adjusted^2 ^OR (95% CI)	Unadjusted OR (95% CI)	Adjusted OR^1^ (95% CI)	Adjusted OR^2 ^(95% CI)

Shops						
Disagree	1.00	1.00	1.00	1.00	1.00	1.00
Agree	1.15 (0.81–1.62)	1.49 (0.95–2.34)	1.73 (1.06–2.84)*	1.08 (0.78–1.50)	1.06 (0.68–1.65)	0.84 (0.52–1.38)
Transit stop						
Disagree	1.00	1.00	1.00	1.00	1.00	1.00
Agree	0.94 (0.66–1.33)	1.30 (0.81–2.08)	1.16 (0.69–1.93)	0.96 (0.68–1.37)	0.69 (0.40–1.19)	0.59 (0.32–1.07)
Sidewalks						
Disagree	1.00	1.00	1.00	1.00	1.00	1.00
Agree	1.04 (0.67–1.61)	1.25 (0.61–2.59)	1.21 (0.54–2.71)	0.93 (0.59–1.44)	1.02 (0.46–2.25)	0.62 (0.26–1.49)
Bicycle facilities						
Disagree	1.00	1.00	1.00	1.00	1.00	1.00
Agree	1.12 (0.79–1.59)	1.12 (0.74–1.70)	0.90 (0.57–1.42)	1.31 (0.93–1.84)	1.24 (0.80–1.94)	1.12 (0.69–1.80)
Free or low cost						
Disagree	1.00	1.00	1.00	1.00	1.00	1.00
Agree	1.09 (0.74–1.61)	0.97 (0.60–1.57)	0.86 (0.51–1.45)	1.53 (1.05–2.24)*	1.43 (0.86–2.38)	1.18 (0.67–2.05)
Crime						
Disagree	1.16 (0.73–1.85)	1.20 (0.70–2.04)	0.83 (0.46–1.50)	1.32 (0.94–1.86)	1.40 (0.90–2.15)	1.50 (0.94–2.40)
Agree	1.00	1.00	1.00	1.00	1.00	1.00
Traffic						
Disagree	0.91 (0.60–1.39)	0.94 (0.57–1.54)	0.87 (0.51–1.50)	1.04 (0.70–1.54)	1.06 (0.66–1.72)	0.98 (0.58–1.67)
Agree	1.00	1.00	1.00	1.00	1.00	1.00
Seeing people active						
Disagree	1.00	1.00	1.00	1.00	1.00	1.00
Agree	1.17 (0.81–1.68)	1.20 (0.78–1.83)	1.17 (0.74–1.85)	1.50 (1.01–2.20)*	1.78 (1.08–2.91)*	1.59 (0.93–2.71)
Interesting things						
Disagree	1.00	1.00	1.00	1.00	1.00	1.00
Agree	1.83 (1.29–2.58)**	1.94 (1.302.88)**	1.65 (1.08–2.53)*	1.20 (0.85–1.69)	1.16 (0.76–1.77)	0.94 (0.59–1.50)
Easy access						
Disagree	1.00	1.00	1.00	1.00	1.00	1.00
Agree	2.40 (1.46–3.93)***	2.49 (1.43–4.32)**	1.82 (1.00–3.31)*	2.63 (1.72-	2.72 (1.56-	1.61 (0.87–2.96)

* p < .05., **p < .01., ***p < 0.001.

Note^1^: Adjusted by age, education, income, and location (urban vs. rural).

Note^2^: Adjusted by age, education, income, location, and self-efficacy.

Perceptions of easy access to places for PA were associated with higher levels of LTPA in both men and women (OR = 2.40 and OR = 2.63, respectively). For men, the relationship was still statistically significant after adjusting for sociodemographic variables (OR = 2.49) and self-efficacy (OR = 1.82). For women, the association was still significant after adjusting for sociodemographic variables (OR = 2.72), but not after self-efficacy was entered in the model.

## Discussion

This study explored gender differences in perceptions of the physical environment and in environmental correlates of LTPA in a representative sample of adults from the province of Alberta. Our results are consistent with a growing body of literature that suggests the existence of a link between several dimensions of the perceived environment and self-reported LTPA [20,31]. Further adding to this literature, our analyses show that other studies may have overlooked an important moderating effect of gender in the relationship between the perceived environment and LTPA.

Unlike previous work concerned with gender differences in the association between PA and environmental variables [26], women and men in our study did not differ, at least to a statistically significant degree, in perceptions of traffic as a potential problem for walking. Consistent with this research [26], women were more concerned than men about the safety of walking at night. Nevertheless, perceptions of low safety were not significantly associated with lower levels of LTPA in women, which is in-line with results from previous studies assessing specific correlates of PA in women [45-47].

Several interesting differences emerged between women and men in terms of the dimensions of the perceived environment that were linked with increased LTPA participation. The perceptions that "there are many interesting things to look at while walking" and that "there are many places to buy things within easy walking distance from home", were significantly associated with higher levels of LTPA in men but not in women. This is somewhat inconsistent with a recent study [24] that found both men and women reporting positive changes over time in aesthetics and convenience in their neighbourhood were at least twice as likely to increase their walking. Likewise, another recent study [26] found that convenience (i.e., availability of shops within walking distance) was positively associated with high levels of walking (>150 mins/week) among women. One possible explanation for our findings is that perceived environments indeed have a differential impact on LTPA according to gender. Another possible explanation is that the role of perceived environment in relation to behaviour may be moderated by cultural and national factors that vary by country and region.

Conversely, since men reported higher levels of LTPA than women, interpretations that PA can affect perceptions of the environment are plausible as well [45]. Offering some support for this hypothesis, we found that men were higher than women in perceptions of easy access to places for PA and of availability of places to buy things within easy walking distance from home. Thus, it could be suggested that individuals who are physically active may be more likely to perceive that they have access to places where they can get PA, or to places where they can buy necessary things, and that they can reach by physically active means (e.g., walking) [48]. Alternatively, these findings may have been due to chance, thus replication of them is necessary. In any case, the unexpected results suggest that the interaction between environmental influences and individual difference characteristics may be more complex than previously thought.

Perceiving that one's neighbourhood has several free or low cost recreation facilities was associated with higher levels of LTPA in women but not in men. This finding makes some sense when taking into account that the average annual household income of the women in our sample was lower than that of men. Studies have found that low amounts of leisure-time PA are strongly associated with low income [49,50], low education [51], and low socio-economic status [52,53]. Moreover, the lowest participation rates have been found amongst the poor and women of child-rearing age, many of whom are the same people [54]. In fact, low-income women identify a lack of access to PA in their community as a major factor inhibiting the development of healthy lifestyles for themselves and their families [54]. Thus in the case of our results, having access to places where one can participate in PA for free or at a low cost may have been an incentive (i.e., facilitator) for women to get involved in PA.

Likewise, seeing many people being active in one's neighbourhood was associated with increased levels of LTPA only in women. The positive impact of physically active role models on women's participation patterns has been documented in other studies investigating the influence of characteristics of the neighbourhood environment on PA [45,46]. Bandura's [55] social cognitive theory explains how role modelling can influence behaviour by enhancing an individual's sense of self-efficacy. Women in our sample had lower self-efficacy to overcome barriers to PA than men. Consistent with previous research showing the importance of self-efficacy on women's LTPA [16,47], this may help explain why physically active role models seem to have been more influential for women in our study. However, the circumstance that neither having access to places where one can participate in PA for free or at neither a low cost, nor seeing many people being active in one's neighbourhood reached statistical significance when adjusted by self-efficacy points to an interesting phenomenon. It seems indeed as if self-efficacy to overcome barriers to PA participation "in spite of" an environment that may facilitate or hinder participation was more influential among women in our sample than self-efficacy "because of" a supportive environment (i.e., one in which role models and low cost facilities are available).

Having easy access to places for PA was the environmental dimension most strongly associated with LTPA in both men (even after adjusting for sociodemographic factors and self-efficacy) and women (even after adjusting for sociodemographics). Once again, the influential role of self-efficacy on women's LTPA in our sample was evidenced when adjusting for this variable had the effect of eliminating the statistical significance of the association. Access to places for PA has previously been linked with increased levels of PA in some studies [48,56] but not in others [46,47]. As Huston et al. [48] have discussed, this measure may appear more strongly related with PA simply because it provides a more global indicator of whether or not an individual has access to suitable places for PA, whether in their neighbourhood or not. Alternatively, it could be that individuals who are physically active may be more likely to perceive that they have access to places or facilities where they can get PA [48]. As our study shows, accounting for gender differences (e.g., in self-efficacy levels) may help to partially explain the discrepancy in results across studies assessing the impact of perceptions of access to places for PA. Thus, interventions should be designed to increase women's self-efficacy to participate in PA when environmental supports may not be readily available. Our results also suggest that in order to encourage women to become more physically active, interventions could be designed to increase awareness and use of environmental supports [57] such as places were people can engage at low or no cost in PA while seeing socially and economically similar others doing the same.

This study has several limitations. First, our data is cross-sectional and therefore we cannot make causal inferences. We also relied on a telephone survey format, which may have resulted in some segments of the population (e.g., low income individuals lacking access to telephones) being underrepresented. Additionally, we used self-report measures of both LTPA and perceptions of the physical environment. It is thus possible that the data may have been subject to biases. Since it is not clear yet whether the actual or perceived environment is more influential [9,45,58], it is important that future studies include assessments of both dimensions in their designs.

## Conclusion

To date, few studies have explored gender differences in perceptions of the environment and how these differences may have an impact on PA patterns of individuals. We found several gender relevant differences in the ways individuals perceive their physical environments and on the dimensions of these environments that are associated with LTPA. Thus, the results from this study add to the knowledge base of gender differences in environmental correlates of PA. Our findings provide further support for the need of using models in which gender is treated as a potential moderator of the link between the perceived environment and PA. Further, these results suggest the possibility of designing interventions to increase PA that address differentially both structural (e.g., income disparities) and social cognitive (e.g., self-efficacy) factors typically associated with gender.

## Competing interests

The author(s) declare that they have no competing interests.

## Authors' contributions

EGB participated in the conception and design of the study, performed the statistical analyses and drafted the manuscript. JCS participated in the conception and design of the study, took a coordination role, and helped to draft the manuscript. KRM coordinated the design of the survey and helped to draft the manuscript. All authors read and approved the final manuscript.
